# Risk of childhood trauma exposure and severity of bipolar disorder in Colombia

**DOI:** 10.1192/j.eurpsy.2023.461

**Published:** 2023-07-19

**Authors:** F. Guillen, J. F. Galvez-Florez

**Affiliations:** ^1^School of Medicine, Universidad Simon Bolivar; ^2^Center for Clinical and Translational Research, La Misericordia Clinica Internacional, Barranquilla, Colombia

## Abstract

**Introduction:**

Bipolar disorder (BD) is higher in developing countries. Childhood trauma exposure is a common environmental risk factor in Colombia and might be associated with a more severe course of bipolar disorder in Low-Middle Income-Countries. We carried out the first case-control study (114 BD patients and 191 controls) in Colombia using a structural clinical interview and the Childhood Trauma Questionnaire-Short Form (CTQ-SF) to describe the prevalence and association between trauma exposure during childhood with a severe course of illness in a sample of BD patients.

**Objectives:**

to describe the prevalence and association between trauma exposure during childhood with a severe course of illness in a sample of BD patients.

**Methods:**

A case-control study (114 controls versus 191 controls) that assessed outpatients between 18 and 65 years old, at a teaching hospital in Barranquilla, Colombia was carried-out. All participants were assessed with the SCID-5-CV, the Young Mania Rating Scale (YMRS), and the Bipolar Depression Rating Scale (BDRS). Additionally, exposure to childhood trauma was assessed using The Childhood Trauma Questionnaire-Short Form (CTQ-SF). The CTQ-SF is a brevity 28-item Likert-type, with a five-factor structure: emotional abuse EA, physical abuse PA, sexual abuse SA, physical neglect PN, and emotional neglect EN, self-administered instrument in order to assess multiple types of trauma during childhood.

We generate an outcome variable named severe bipolar disorder defined by course severe of bipolar disorder as the presence of any clinical indicator of severity, previously delimited by the research team (early-onset, rapid cycling, ideation or suicide attempt, or 3 or more hospitalizations per year). Also, we carried out bivariate and regression analyses with each clinical indicator of severity as an outcome.

**Results:**

Cases included 61.4% BD type I and 38.6% BD type II. The median age was 31.5 years (IQR, 75-24) for BD patients and 31 years old (IQR, 38-24) for healthy controls. A higher prevalence of childhood trauma was evidenced in cases compared to controls.

*Multivariate logistic regression model in severe bipolar disorder*

**Table:** 

		**Severe Bipolar Disorder**						
**Variable**		***B***	***SE***	***OR***	**95% CI**	***p* value**	***p* model**	***R***^***2***^
Emotional Abuse	0.83	0.36	2.30	1.75	3.03	<0.001	<0.001	0.10
Physical Abuse	1.07	0.43	2.92	1.54	5.53	<0.001	<0.001	
Sexual Abuse	1.61	0.44	5.04	4.73	5.36	<0.001	<0.001	
Physical Neglect	0.28	0.49	1.32	0.93	1.87	0.117	<0.001	
Emotional Neglect	1.24	0.38	3.45	2.28	5.23	<0.001	<0.001

**Conclusions:**

This is the first association study between childhood trauma exposure as a higher risk for a severe course of illness in BD patients in Colombian. Our findings highlight the importance of screening and evaluating childhood trauma exposure during the course of BD patients.

**Disclosure of Interest:**

None Declared

